# Understanding the emergence of suicidal thoughts and behaviors in adolescence from a brain and behavioral developmental perspective

**DOI:** 10.1038/s41386-025-02168-2

**Published:** 2025-07-25

**Authors:** Charles P. Lewis, Bonnie Klimes-Dougan, Paul E. Croarkin, Kathryn R. Cullen

**Affiliations:** 1 Department of Psychiatry and Behavioral Sciences, University of Minnesota Medical School, Minneapolis, MN USA; 2 Masonic Institute for the Developing Brain, University of Minnesota, Minneapolis, MN USA; 3 Department of Psychology, University of Minnesota, Minneapolis, MN USA; 4 Departments of Pediatrics, Pharmacology, and Psychiatry and Psychology, Mayo Clinic, Rochester, MN USA; 5 Department of Neuroscience, University of Minnesota, Minneapolis, MN USA

**Keywords:** Human behaviour, Development of the nervous system, Risk factors

## Abstract

Suicide is a leading cause of death in adolescents, and a spectrum of suicidal thoughts and behaviors (STBs) is common among teenagers. Adolescence is a transitional period marked by critical brain changes, coinciding with major changes in how teenagers regulate emotions and impulses, as well as in how they understand themselves and interact with others. We review neuroimaging evidence supporting a developmental conceptualization of suicide risk, focusing on neural changes associated with key developmental tasks of adolescence. Functional and structural imaging studies have implicated medial prefrontal, cingulate, dorsolateral prefrontal, orbitofrontal, and frontolimbic circuitry changes in youth with STBs. There is emerging evidence that psychotherapeutic and neuromodulatory interventions can engage these brain processes and modify behavior in at-risk youth. We argue that harnessing these techniques more specifically by using targeted approaches aimed at enhancing emotion regulation, impulse control, positive identity development, and healthy social functioning is a promising way forward for reducing suicide risk in teens. Continued investigation into neural trajectories of suicidality in adolescence is critical for developing more effective risk assessment and treatment approaches to aid suicidal youth in navigating adolescent development and transitioning successfully to adulthood.

## Introduction

What is it about the teenage years that causes so many youth to consider, attempt, or die by suicide? Suicide is the second most common cause of death among adolescents and young adults in the United States [1, 2] and a leading cause of teen mortality worldwide [3]. Suicide rates among youth have increased substantially in the past two decades [4–6]. Risk factors for death by suicide include a spectrum of suicidal thoughts and behaviors (STBs), as well as nonsuicidal self-injury (NSSI) [7]. Recent U.S. and global data, although varying by region and specific population, highlight the striking prevalence of STBs among youth: 14.2–22.6% considered suicide, 7.5–17.6% planned an attempt, and 4.5–15.8% attempted suicide [8–10]. NSSI is also remarkably common in adolescents [11–13]. Although STBs do occur in children [14], the prevalence of STBs and other behaviors that increase suicide risk, such as NSSI [15], increase substantially during the late pre-teen and early teenage years, with steady increases through adolescence [16]. This suggests a critical developmental window for the emergence of factors that increase risk for suicide. Understanding the neurodevelopmental processes that underlie this increased risk for suicide in adolescents is crucial to guide prevention and intervention efforts.

Adolescence is characterized by unique opportunities for positive growth, including adaptive exploration, developing social competence, and specialized learning, with maturational brain changes supporting the navigation of these opportunities in healthy development [17, 18]. Research using resting-state functional magnetic resonance imaging (fMRI) has revealed meta-organizational brain changes that occur between childhood and adulthood, involving increasingly distributed network architecture [19]. Against this backdrop of developmental processes in the adolescent brain, many mental health problems first emerge during the teenage years [20]. Paus and colleagues [21] outlined a theoretical neural basis for the co-occurrence of these phenomena, considering mechanistic elements of neural development during adolescence. They proposed that synaptic pruning, myelination, and reshaping network connections contribute to a vulnerability to the onset of mental illness in adolescence by virtue of the instability associated with these processes, with the idea that “moving parts get broken” (p. 9) [21]. Brain development mechanisms have been posited as central to the onset of mental health disorders that commonly arise in adolescence [22, 23].

In this review, we take a developmental psychopathology approach [24] to understand suicide risk in adolescents. A previous review by Auerbach and colleagues [25] provided an excellent summary of results from brain imaging studies examining neural correlates of STBs and NSSI in youth. Beyond providing an update from recent neuroimaging studies in adolescents with STBs, here we take a more developmentally-focused perspective to neural mechanisms that underlie key developmental tasks of adolescence and form the emotional, cognitive, and social context in which STBs occur in youth. Although systemic, environmental factors have been implicated in the risk for STBs in youth [26–28], particularly in minoritized populations [29, 30], our review focuses on the individual-level developmental processes by which adolescents navigate the stressors they encounter. Through this developmental psychopathology lens, we acknowledge the importance of understanding typical developmental processes as we explore the probabilistic—but not deterministic—perturbations that take place in the context of adolescent STBs [31]. Although NSSI frequently precedes suicidal behavior [32] and increases risk for future suicide attempts [33], there are important distinctions phenomenologically [34]—and potentially neurally [35]—between NSSI and thoughts or behaviors in which suicide is the intended outcome. Considering the relevance of NSSI to suicide risk, we review neuroimaging literature on both NSSI and STBs in relation to adolescent developmental constructs, but note that brain mechanisms may not be uniform across the broad spectrum of NSSI and STBs.

We propose that neural systems involved in cognitive processes and behavioral regulation unique to adolescence are critical to understanding the emergence of STBs in this age range. In particular, we highlight neuroimaging research that has investigated brain systems underlying key developmental tasks of adolescence that are theoretically relevant to suicide risk: how adolescents regulate negative emotions, how they inhibit impulses in the context of negative emotions, how they perceive and understand themselves and their identities, and how they navigate relationships. This literature provides a framework for discussing neuroscience-informed intervention opportunities pertaining to these tasks, and how continued imaging and other brain-based research into neural systems that undergo rapid change and maturation during adolescence will lead to more developmentally-sensitive approaches to risk assessment and intervention in youth with STBs.

## Disruption in adolescent developmental processes in suicidal thoughts and behaviors

### Regulation of negative emotional experiences

Suicide often occurs in the context of unbearable emotional anguish or psychological pain, which Shneidman termed *psychache* [36]. Research on adolescent survivors of suicide attempts has shown that negative emotion (e.g., depression, anger) [37] and emotional pain [38] are primary drivers of suicide attempts. Adolescence is a time when threat perception systems are heightened, yet the drive for exploration and novelty often surpasses the safety of the familiar [39]. Homeostatic threat evaluation and novelty-seeking confer evolutionary advantages. However, for those at heightened risk for suicide, there is a high burden of negative emotions, like sadness, anger/irritability, worry, and fear. Both the persistence of negative affect and increased emotional lability may unduly tax threat processing systems during this developmental period, requiring more neural resources to regulate emotion [40]. The capacity to navigate negative emotions effectively is still developing during the teen years; acquiring these regulatory skills is necessary for resilience to stressors during adolescence and throughout adulthood.

Frontolimbic neural structures are centrally implicated in the regulation of negative emotions. The prefrontal cortex (PFC) and cingulate cortex, regions crucial to emotion regulation, undergo systematic change from childhood through adulthood [41]. Key limbic structures, particularly the amygdala, have reciprocal connections with the PFC and other limbic regions and are prominent in threat and negative emotion processing [42, 43]. Adjacent regions (e.g., insula, hippocampus, and temporal gyri) are involved in identifying and contextualizing salient emotions. Research over the past three decades has begun to elucidate developmental mechanisms for frontolimbic systems, including cortical pruning and subtle increases in amygdala volume into early adulthood [44, 45].

Developmental perturbations of frontolimbic structures have been associated with suicide risk in several structural imaging studies in youth with STBs. In a large multisite adolescent study, prior suicide attempt was associated with lower frontal pole surface area [46]. Adolescents with depression and suicide attempts have decreased volume in the right superior temporal gyrus, which is involved in auditory, language, and emotional response processing, compared to healthy controls, although not to depressed non-attempters [47, 48]. Another study by Vidal-Ribas et al. [28] examined data from 9–10-year-olds from the Adolescent Brain Cognitive Development (ABCD) Study [49], which has enrolled more than 11,000 children at 21 sites in the United States and collects multimodal neuroimaging and clinical phenotyping data, beginning at age 9–10 years and continuing throughout adolescence. The authors found that reduced cortical thickness of the superior temporal sulcus was associated with caregiver-reported STBs [28]. In a study of youth with bipolar disorder, those with prior suicide attempts had reduced orbitofrontal cortex, hippocampus, and cerebellar volumes, as well as reduced ventral frontal, right cerebellar, and uncinate fasciculus white matter integrity [50]. Further research is needed to determine whether neural processes underlying STBs in youth include cortical pruning and structural mechanisms implicated in depressive symptoms and severe psychosocial stress, such as diminished amygdala volume [51, 52] and accelerated cortical thinning [53]. In addition, longitudinal work is needed to determine whether structural differences represent increased risk for STBs via neural development trajectories that precede the onset of STBs, or whether the stress of suicidal events induces structural changes in developing emotion regulation systems.

Functional imaging studies, particularly those using emotion-processing tasks, have yielded critical understanding of key developmental threat processes and atypical patterns of emotion regulation in frontolimbic systems [54]. Activation of these systems is enhanced during the teenage years; for example, the magnitude of amygdala activation in response to emotional facial stimuli is greater during adolescence than childhood [55] or adulthood [56]. The emotional valence of stimuli is also relevant to amygdala response; in populations with depression and NSSI, amygdala hyperactivation has been observed with negative stimuli, such as angry and fearful faces [57–59], whereas positive stimuli were associated with hypoactivation [57]. Other studies have sought to distinguish differential activation patterns in depressed teens with and without STBs. Pan et al. [60] found that depressed adolescents with suicide attempts had significantly greater activation in the anterior left dorsolateral prefrontal cortex (DLPFC), anterior cingulate cortex (ACC), and primary sensory cortices when viewing angry faces than depressed adolescents without attempts. In another study, the same group examined activation in response to another implicit emotional-faces task, finding that adolescents with depression and prior suicide attempts demonstrated less activation in the right DLPFC than controls in response to all emotional faces, although they did not differ from depressed teens without attempts [61]. However, using an explicit emotion regulation task, Miller et al. [62] observed that depressed adolescents with suicidal ideation had heightened DLPFC activation compared to those without suicidal ideation; they also had less DLPFC, temporoparietal junction, and cerebellum activation during passive viewing of negatively-valent images compared to controls, which remained significant when controlling for depression and exposure to adversity.

Functional connectivity patterns involving structures and networks involved in emotion regulation may provide additional insights into developmental changes in emotion regulation during adolescence that impact suicide risk. Compared to children, adults show stronger positive connectivity between PFC and amygdala [63], and preliminary evidence suggests a shift from positive to negative connectivity during adolescence [64]. Although some adolescent studies have found altered connectivity between frontolimbic regions in youth with depression and NSSI [65, 66], few studies have employed analyses of functional connectivity in youth with STBs. In their previously mentioned study, Pan et al. [60] noted decreased dorsal ACC–insula functional connectivity in adolescents with depression and prior suicide attempts compared with depressed nonattempters. In the aforementioned study by Johnston et al. [50], functional imaging also revealed that youth with bipolar disorder and prior suicide attempts had reduced connectivity between the amygdala and left ventral and right rostral PFC compared to bipolar nonattempters; amygdala–left ventral PFC connectivity correlated negatively with attempt lethality. Another structural and functional MRI study in untreated suicidal youth found that those with high suicide risk had smaller right frontal pole surface area and reduced right frontal pole–bilateral inferior frontal cortex functional connectivity than youth with low risk [67]. Other innovative paradigms to measure threat and distress responses are being developed [68, 69], although small samples impede the ability to make strong conclusions about connectivity patterns. Although limited, the extant literature points to PFC–limbic connections—critical to regulating negative emotional experiences—being weaker in youth with versus without STBs.

In summary, emerging literature on regulation of negative emotions in adolescent STBs has implicated brain regions largely within, but also beyond, the frontolimbic network. These findings partially map onto the broader literature on neural correlates of affect regulation in youth with other risk factors for suicide (e.g., depression and/or NSSI) [35]. Further research is needed to understand neural signatures of disrupted emotion regulation systems in youth with the most severe STBs, such as suicide attempts or death by suicide. This represents a population with considerable challenges to evaluate empirically, but one that is critically important to understand in order to prevent deaths by suicide [70].

### Emotion-relevant impulse control

Developing the ability to inhibit impulses and regulate behavior is a critical maturational task of adolescence. Impulsivity in teens has been implicated in risk for suicide attempts and other STBs [71–73]. Various experimental models of inhibitory control have been developed [74], including Go-No/Go, Stop-Signal, Delay Discounting, and Continuous Performance Tasks [75–79]. Some impulsivity paradigms have shown associations with STBs, although mixed findings (e.g., [80–84]) point to the heterogeneity of processes underlying impulse control. Contemporary theories highlight dynamic, context-dependent aspects that contribute to a fluid capacity to inhibit impulses and regulate behavior, leading to the development of assessments to capture momentary fluctuations [85] and behavioral tasks to simulate contextual influences such as emotional state [86]. Inhibiting action-oriented urges specifically when experiencing negative emotions and suicidal thoughts has been studied increasingly in relation to suicidality [87]. The tendency to demonstrate rash behavior during intense emotions was termed *urgency* in Whiteside and Lynam’s model [88], later classified into positive and negative urgency depending on the affective valence [89]. There is evidence for a developmental trajectory of negative urgency, which increases during early adolescence before plateauing around age 16 [90]. Negative urgency has been studied across a broad range of neuropsychiatric conditions in youth [91], and prior research has found associations between negative urgency and STBs across a range of ages from late childhood to emerging adulthood [92–95]. Early deficits in emotion-relevant impulse control may constitute part of a trajectory of risk; elevations in negative urgency and other impulsivity measures by age 9–10 have been found to predict later STBs, including attempts [96, 97]. In addition, difficulty inhibiting thoughts and urges related to self-harm and suicide may play a role in the transition between suicidal ideation and suicidal actions during developmentally-typical intense emotional experiences and still-maturing affect regulation. In a high-risk sample of adolescent inpatients (13-19 years), Auerbach et al. [98] found that while the influence of feelings on self- and future-thoughts correlated with suicidal ideation, impulsive reaction to emotions was associated with suicide attempts.

Despite emerging evidence for negative urgency increasing the risk for STBs in young populations, little research to date has examined neural mechanisms of emotion-relevant impulsivity constructs in youth with STBs. Neuroimaging research in non-suicidal adolescent populations has found associations between negative urgency and orbitofrontal, dorsolateral prefrontal, and lateral temporal cortical volumes [99]. Additionally, negative urgency has shown different patterns of volumetric associations based on sex [100] and body composition [101, 102] in youth, suggesting variable trajectories of neural development as teens progress through puberty and adolescent growth. The degree to which these observations from other populations apply to brain mechanisms of negative urgency in youth with STBs is unclear. The superior temporal structures, in which reduced cortical thickness was found to be associated with STBs in 9–10-year-olds in the previously noted study by Vidal-Ribas et al. [28], have also been implicated in impaired impulse control in older suicide attempters [103]. In another ABCD Study analysis, Wiglesworth et al. [104] examined rapid-response impulsivity in Native American participants (age 9–10 years), finding that negative urgency was associated with greater odds of having experienced suicidal ideation. In this sample, activation of the orbital right inferior frontal gyrus during an emotionally-neutral response inhibition task correlated with negative urgency scores, although not with suicidal ideation. The authors noted that behavioral paradigms simulating impulse control in negative valence conditions may demonstrate different activation profiles than emotionally-neutral ones, and that neural mechanisms of negative urgency may appear later in adolescent brain development when negative urgency increases [104]. Other ongoing multimodal work [105], based on prior observations of impaired cortical inhibition in adolescents with suicidal behavior [106] and ideation [107], aims to examine neurophysiologic mechanisms of emotion-dependent impulse control deficits in suicidal youth, using transcranial magnetic stimulation paired with electroencephalography and functional imaging (an emotional response inhibition task [86]) to measure inhibitory processes in frontal networks over time in youth with STBs.

The evidence for impulse control deficits during heightened negative affect having an effect on the risk for STBs in youth is growing, but the literature on brain networks underlying these deficits is currently nascent. Continued longitudinal work is needed to provide insights into developmental trajectories of negative urgency and its neural mechanisms, and particularly how they change as STBs emerge across adolescence.

### Self-concept and identity development

The decision and action to end one’s own life—to bring an end to the self—likely requires a certain disregard or devaluation of the self as something not worth preserving. This highlights the need to consider the development of self-relevant processes in the context of suicide risk in adolescents. The teenage years have long been associated with the process of exploring and consolidating identity in psychoanalytic literature, particularly Erikson’s [108] influential theories of psychosocial development. The emergence of suicidal thoughts during adolescence coincides with a time notable for identity development and forming one’s sense of self [109, 110], and ongoing maturation of brain regions supporting those functions [111]. Suicidal thoughts are frequently accompanied by feelings of worthlessness [112–114] for children [58] and adolescents [115–118]. As adolescents navigate new challenges in multiple realms (academics, relationships, early work experiences), they explore and discover new aspects of themselves and ways they can fit into the world, iteratively trying on different “possible selves” [119]. The neuroplasticity and remodeling enabling these formative processes may also introduce vulnerability for developing negative self-perceptions, thereby opening a susceptibility to the onset of ideas about suicide related to negative evaluations of one’s evolving roles and identity.

The neural underpinnings of self-referential processing primarily involve medial cortical structures, such as anterior and posterior cingulate cortices, medial prefrontal, orbitofrontal, and parietal cortices, and the retrosplenial cortex [120]. These structures, and the processes they support, undergo significant development across adolescence. For example, studies of typical development suggest that rostral ACC activation during self-relevant word processing increases between late childhood and early adolescence [121] and across mid-adolescence [122]. While a subsequent, larger longitudinal study of girls across the pubertal transition did not demonstrate age- or puberty-related changes in medial cortical activation during self-evaluations, it did find that higher activation in the ventromedial PFC and perigenual rostral ACC was associated with more frequent negative self-evaluations and less frequent positive self-evaluations [123]. This pattern has been observed in studies of later adolescence: a longitudinal study of self-concept and its neural underpinnings in youth ages 10-24 years reported that the mid-adolescent period was notable both for a decline in self-concept positivity and a peak in medial PFC activation during self-evaluations [124]; whereas greater ventromedial PFC activation during self-evaluations was related to greater ill-being and lower social well-being in college freshmen [125].

While these studies in typically-developing young populations have begun to reveal a pattern in which greater activation of medial prefrontal regions during self-evaluation is associated with more negative self-view and ill-being, results from studies that have focused on youth with increased suicide risk have been mixed. Quevedo et al. [126] studied adolescents with varying levels of risk, viewing their own and others’ faces in different emotional contexts during an fMRI paradigm. Adolescents with high suicide risk (current suicidal ideation, history of suicide attempts) showed *lower* activation of medial cortical networks in response to self faces (compared to others’ faces) [126]. A similar pattern was observed in another study of adolescents oversampled for history of NSSI; severity of NSSI was related to lower self-worth, higher frequency and speed of negative self-evaluations, *lower* anterior medial cortical activation during self-evaluation, and *lower* resting-state functional connectivity within the network comprised of medial cortical regions [118]. However, these results diverge from a study reporting that adolescents with depression and NSSI history showed *greater* activation in anterior and posterior cortical midline structures and limbic areas during self-evaluations compared to those without NSSI [127]. Cognitive rumination, which may perpetuate negative self-evaluation in adolescents, also has been linked to default mode network (DMN) structures, including the dorsomedial PFC, dorsal ACC, and insula [128, 129].

Together, the limited extant literature suggests that medial cortical regions mediate self-processing, and that these brain structures and processes develop across the adolescent period. Greater activation in these regions during self-processing may signify more negative self-perception and indicate risk in typically-developing youth; however, in the context of significant psychopathology (e.g., depression, NSSI, and STBs), the activation pattern of midline cortical and other DMN nodes may become more complex. Longitudinal research, potentially utilizing large population-based samples, is needed to understand when and how these deviations emerge, to conceptualize the underlying processes more completely, and answer key developmental questions: whether such changes stem from adaptation to environmental stressors versus neural scarring effects, and whether particular subgroups at risk for STBs have distinct neural patterns underlying negative thoughts about themselves.

### Social development

Emotion regulation, impulse control, and identity development are intertwined with social development, another important task of adolescence that also has been associated with suicide risk. Identity development is increasingly recognized as an inherently social process [130], known to be shaped by relationships with others at both behavioral and neural levels across the adolescent period [131]. Emerging research suggests that peer relationships contribute positively to adolescents’ identity formation [132]. Compared to children, whose self-concepts are more individualistic, adolescents tend to include their social relationships as integral to their own identities [133]. Rooted in early caregiver attachments and intergenerational influences, teens form new and often intense relationships during the adolescent years, which in turn influence their evolving and dynamic sense of self [134, 135]. A longitudinal study of self-views across adolescent development reported that when describing themselves, the negative impact of social comparison was greatest for the mid-adolescent age group [124].

Suicidal thoughts and behaviors often occur in the context of relational disturbances, which are common as teens practice new modes of interacting and form new types of relationships. Although social interactions begin in infancy and continue throughout the lifespan, adolescence is a critical period for social development. Teens typically become increasingly focused on relationships with peers rather than those with their families of origin (“leaving the nest”) [136, 137]. Attaining the ability to create and navigate new social networks is critical for success in adulthood. For some adolescents, new peer relationships may create greater emotional volatility and higher stress than family relationships [138]; conversely, for some teens, family conflict may increase risk for STBs [139]. Although interpersonal threats are commonly experienced in adolescence, some teens face additional threats to physical safety. Relational perturbations may increase risk for STBs through losing a sense of inclusion or belongingness to others, as described in the Interpersonal-Psychological Theory of Suicidal Behavior [140]. Recent work has implicated these constructs in adolescent STBs [141, 142], including a study finding that lack of relational belonging prospectively predicted adolescent suicide attempts [143].

Contemporary social development has considerable complexity, as many adolescents today spend much of their time engaged in digital environments, with interactions occurring through social media platforms [144]. Negative affect and suicide risk may be associated with social media and other digital interactions (e.g., online “influencers” in one’s social circle and those defined algorithmically by platforms) that have become the primary mode of adolescent communication in recent decades [145]. Interpersonal threats in digital milieus may have lasting and pernicious effects during neurodevelopment related to broader social audiences and exposures. Threats to teens’ sense of belongingness in digital communications may occur rapidly and publicly, increasing risk for STBs through negative digital interactions. However, adolescents can also utilize social media to mobilize social support remotely in the face of stressors and conflicts, even when unable to do so via in-person interactions [146].

Substantial neurobiological shifts accompany the social developments of adolescence. PFC maturation facilitates responsible decision-making, planning, impulse control, and self-regulation [147]. Concurrently, limbic pathways become more active with enhanced emotional and reward processing. Often, neural pathways contributing to heightened emotional responses overtake cognitive control and regulation of spontaneous behaviors and drives in adolescent social contexts [148]. This neurodevelopmental tension often fosters heightened emotional reactivity, with notable hyperactivity to negative social cues, risk-taking behaviors, and increased suicide risk during adolescence [149, 150]. Research using a social inclusion/exclusion task (the Cyberball game, [151]) and a response inhibition paradigm (Go/No-Go task) demonstrated that depressed female adolescents with previous suicide attempts had decreased left insula activation with social inclusion and higher right middle prefrontal gyrus activation during social exclusion, underscoring that neural changes in social perception and response inhibition may create unique vulnerabilities for STBs in adolescent girls [152]. In another study [153], depressed adolescents with high suicidal ideation showed reduced activation in caudate, putamen, insula, and ACC, and in precentral, postcentral, superior temporal, and medial frontal gyri, during the Cyberball task. Adolescents with suicide attempts showed higher activity in ACC and in superior and middle frontal gyri than other adolescents [153]. The frontolimbic circuitry discussed earlier in relation to emotion regulation has also been implicated in research examining social stimuli. Teens with recent suicide attempts demonstrated increased left amygdala–rostral ACC connectivity during self-face recognition [154], suggesting that activation of threat- and fear-related systems in the perception and processing of social stimuli (or, alternatively, self-perception) may contribute to suicide risk.

In summary, adolescents’ social development—particularly with new peer relationships—intersects with emotional, impulse control, and self-perception processes, which may have unique features in contemporary digital social interactions. Emerging but limited neuroimaging literature implicates frontolimbic structures in social exclusion, suggesting that altered social processing may play a role in risk for adolescent STBs.

### Common circuitry among developmental tasks of adolescence?

Contemporary research on neural correlates of emotion regulation, impulse control, identity development, and social development in youth with and without STBs shows considerable convergence on medial frontal brain structures. To date, emotion regulation systems have accumulated the greatest amount of evidence for involvement of networks involving the medial frontal cortex. These structures, including the medial PFC and ACC, play crucial roles in evaluating environmental stimuli and determining relevance to oneself [155]. Medial frontal structures are posited to mediate inputs from both limbic (threat-based responses) and lateral prefrontal (executive/evaluative) systems, permitting assessment of stimuli and assignment of affective valence, as well as directing attention, both key in salience processing [120, 156–158]. Moreover, the medial PFC is a major node of the DMN, which has been implicated in self-referential thinking, social cognition, internal narratives, and identity formation [159, 160].

A potential explanation of this common involvement of midline cortical structures in affect regulation, emotion-relevant impulse control, self-concept, and social functions is that these processes themselves are interdependent. Effective social functioning requires that one recognize and modulate one’s own emotions, inhibit urges in moments of heightened emotion, and reflect on and project a coherent sense of identity (even as it evolves and changes). Some evidence in the adolescent literature supports the interdependence of these processes, or complex moderation relationships between them. For example, in adolescents and young adults with NSSI, negative urgency was associated with impaired emotion regulation [161], and moderated the relationship between negative emotions and NSSI [162]. Alternatively, distinct cognitive, emotional, and behavioral functions may utilize shared neural infrastructure, which may depend on context more than the specific task [163]. Functional networks show significant individual variation and are relatively less influenced by task state or day-to-day physiologic changes [164]. Multidimensional profiles, or “functional fingerprints”, reveal the diversity of processes utilizing a particular structure, region, or network [165], which vary considerably throughout the brain. Prior work has demonstrated substantial functional diversity in networks involving medial PFC [165], and recent functional neuroimaging literature has highlighted the diversity of individual differences in brain networks that develop during adolescence.

Human brain development consists of an ordered, hierarchical pattern, proceeding from sensorimotor areas (completed in childhood) to association cortices that mediate complex emotional, social, introspective, and executive functions (with ongoing development through the teenage years and into the twenties) [166]. Functional variability between individuals increases in the later-developing, more plastic association areas, but the heterogeneity in these regions may also increase potential for psychopathology, including STBs [166]. Personalized topographical maps of functional networks reflect individual prioritization of cognitive processes; by the pre-teen years, individual topography predicts cognitive performance, with greater predictive ability for higher-order functions [167]. Functional imaging approaches that yield individualized trajectories of neural network topography will enhance the understanding of complex, individually-heterogeneous processes such as emotion regulation, impulse control, self-reflection, and social functioning, and potentially identify topographical patterns indicating increased risk for—or reduced vulnerability to—suicidal behavior.

## Interventions rooted in developmental processes

The growing body of research into neural mechanisms of STBs in adolescents raises intriguing opportunities for clinical applications rooted in the neurobiology of critical developmental processes to prevent attempts and deliver more effective treatments to youth with STBs [168]. Mounting evidence shows that not only does the brain impact behavior, but behavior impacts the brain [169]. These bidirectional processes may have important implications both for the plasticity of adolescent brain development and for psychotherapy interventions. Some psychotherapy approaches have strong clinical evidence for treating adolescent depression and self-harm, but most are still awaiting evidence of links to key neural processes.

The ability to alter the functioning of brain networks involved in emotion regulation, impulse control, identity, and social functioning also suggests avenues for developmentally-informed somatic treatment approaches. Noninvasive neuromodulatory techniques, such as transcranial magnetic stimulation (TMS), deliver focal energy to nodes that induce functional changes throughout widespread cortical networks. TMS and other noninvasive electromagnetic brain stimulation techniques are thought to alter the excitatory–inhibitory balance of neural networks, engaging γ-aminobutyric acid (GABA) and glutamate systems and inducing processes such as long-term potentiation (LTP) and long-term depression (LTD) [170–172]. Clinical antidepressant effects from TMS delivered to the DLPFC may involve altering the connectivity of DLPFC, ACC, insula, and salience network nodes, while other stimulation approaches may have different patterns of connectivity changes [173]. Stimulating the adolescent brain also may have distinct effects from those observed in adults. Early mechanistic work found different patterns of excitatory–inhibitory functioning (measured via TMS-evoked potentials) in teens with depression compared to adults [174], with adolescents showing impaired glutamatergic facilitation. Additionally, a recent TMS clinical trial indicated that depressed adolescents may respond favorably to different stimulation frequencies with therapeutic applications of TMS [175]. An established intervention for depression in adults for nearly two decades, TMS has gained increasing evidence supporting its use in adolescents with depression [176, 177] and received U.S. Food and Drug Administration clearance for ages 15+ in 2024 [178]. Research in adolescents has suggested that TMS also can reduce suicidal ideation, although this effect has been difficult to disentangle from improvement in other depressive symptoms in clinical trials designed to measure antidepressant response [179, 180], and teens with high suicide risk have been excluded from most TMS trials for depression. Few studies have examined effects of TMS and other neuromodulatory interventions on the developmental processes discussed in this review, but preliminary research suggests that noninvasive stimulation may alter emotional, impulse-control, and other functions in ways that could be therapeutically useful.

While more research in youth with STBs is needed to solidify findings regarding brain-based interventions in relation to developmental processes, these insights reveal potential avenues for future, developmentally-sensitive interventional research and rigorous mechanistic clinical trials. Here, we review evidence for psychotherapeutic and somatic treatment approaches that intersect with emotional regulation, impulse control, and social functioning; and, when available, we discuss evidence for their neural mechanisms and effects on risk for STBs.

### Emotion regulation interventions

Interventions bolstering teens’ regulation of intensely negative emotional experiences have received the greatest attention among the developmental processes in our review. Early evidence points to frontolimbic connectivity and hypothalamic–pituitary–adrenal reactivity as predictors of treatment response in psychotherapy approaches used commonly in youth with STBs [181]. Established treatments such as cognitive behavioral therapy (CBT) increase activation of PFC areas when participants are exposed to emotional stimuli [182], enhancing regulation of negative emotion. Dialectical behavioral therapy (DBT) specifically targets affect regulation and currently has the strongest evidence base for reducing suicidal behavior and other self-harm in youth [183]. For adolescents, emotion regulation was found to mediate the relationship between DBT and self-harm remission [184], but skills taught in DBT target a broader range of processes that may have implications for identity development, impulsivity, and social functioning. Potential reasons for its success in reducing STBs include that DBT does not derive from a single theory, and that it is unique in assembling a wide range of skills that can address the potential heterogeneity of developmental processes that place some youth at high risk for self-injury and suicide. Currently, there are innovative efforts to deliver DBT to high-risk youth via novel approaches, such as an intensive DBT program utilizing smartphone-based skill content, passive monitoring, notifications, and between-session clinician support [185]. Among adolescents who underwent psychotherapy (including DBT), more strongly anticorrelated amygdala–medial PFC connectivity at baseline was associated with reduction in NSSI and suicidal ideation [186]. fMRI studies in adults also have found DBT to reduce amygdala [187, 188] and ACC [188] activity.

Some interventions have focused on helping parents and teachers to scaffold adolescents’ developing emotion regulation skills via interpersonal relationships. Tuning in to Teens [189] and Healthy Emotions and Relationships with Teens—A Guide for Parents (HEART-P) [190] target parents’ emotional socialization and parent–adolescent attachment. Family-based treatments for suicidal youth may be particularly impactful for teens who have highly conflictual relationships with their parents, and for those from traditionally underserved populations [191].

A meta-analysis of adult studies found that TMS had significant effects on down-regulating emotion, particularly with right ventrolateral prefrontal stimulation [192]. Although not designed to target emotion regulation specifically, 10 Hz TMS delivered to the left DLPFC in a clinical trial of adolescents with depression increased amygdala and left DLPFC volumes over 6 weeks of treatment [193], suggesting potential utility in modulating threat-perception and negative affect regulation systems. These findings are reminiscent of DLPFC volumetric and functional connectivity changes observed in studies of depressed adolescents treated with selective serotonin reuptake inhibitors (SSRIs) [194].

Psychopharmacologic interventions may also alter emotion regulation. SSRIs inhibit the 5-HT transporter, causing early increases in synaptic serotonin and gradual changes in both sensitivity of serotonin autoreceptors and enhanced glutamate release, leading to increased neural excitability [195]. Long-term alterations in expression of serotonin receptors and neurotrophic factors are thought to underlie increased plasticity, network efficiency, and limbic network connectivity, enabling greater adaptability to environmental stress and emotionally-salient stimuli [195]. Widely used in the treatment of youth (and adults) with depression [196], some evidence supports an effect of SSRIs on emotion regulation processes. In a transdiagnostic adult sample, 12 weeks of SSRI treatment improved emotion regulation, although less so than CBT [197]. Reductions in ACC, amygdala, insula, and thalamus activation during an emotional processing task after 7 days of treatment with escitalopram mediated the antidepressant response at 6 weeks in depressed adults [198]. By contrast, paroxetine increased left DLPFC and supplementary motor area activation during an emotion regulation task, while less PFC recruitment during the task at baseline predicted greater improvement in posttraumatic stress symptoms with the medication [199]. The utility of SSRIs for reducing suicide risk by enhancing developing emotion regulation processes and understanding the specific brain systems engaged will require further research in adolescents.

### Impulse control interventions

To our knowledge, no psychotherapeutic studies have specifically targeted emotion-related impulsivity in adolescents with STBs. However, preliminary research with other populations has suggested areas that may be the focus of future interventions. Negative urgency appears to be modifiable by psychotherapeutic interventions, with reductions in negative urgency observed in patients undergoing residential treatment for substance use disorders [200]. A cognitive control training intervention also produced sustained reduction in emotion-related impulsivity in adults [201]. In a landmark study [202], conducted with first- and second-grade students in low-income schools, suicidal ideation by ages 19–21 was reduced in those whose early elementary classrooms participated in the Good Behavior Game, a program targeting skills for self-regulation of behavior within a peer context [202].

Some investigations have examined neuromodulation outcomes on impulsivity, although rarely as a primary outcome, and typically in adult populations. A meta-analysis of studies with healthy adults found that TMS modulates motor and temporal impulsivity domains, but not reflection impulsivity (premature decision-making) [203]. Transcranial direct current stimulation (tDCS), a noninvasive technique for altering brain function via weak electrical current applied to the scalp, has been employed in adult studies aiming to enhance impulse control, typically with electrodes placed over the DLPFC. Although some results have been promising, the literature is inconsistent, potentially due to heterogeneous study designs and measures, stimulation parameters, and populations [204]. A recent meta-analysis of randomized controlled trials in persons with psychiatric conditions found that neither TMS nor tDCS had significant effects on impulsivity [205]. Further work is needed to examine how emotional context impacts the effects of neuromodulation on impulse control, and whether it can be enhanced effectively in the adolescent brain.

Stimulant medications increase corticostriatal synaptic dopamine and norepinephrine, and have been demonstrated to have widespread but regionally-variable, task-dependent effects on activation in the PFC, anterior and posterior cingulate, insula, amygdala, orbitofrontal and parietal cortices, striatum, thalamus, and cerebellum [206]. Stimulants are commonly prescribed as treatment for impulsive behaviors in attention-deficit/hyperactivity disorder (ADHD), and there is evidence that stimulants reduce suicide risk in retrospective analyses of large samples from insurance data and national databases. Among adult military veterans with ADHD, stimulants reduced suicide mortality [207]. In a within-individual analysis of longitudinal registry data [208] in adults with borderline personality disorder, ADHD medications were the only pharmacologic treatments that reduced risk of attempts or suicide deaths. Another study [209] found that stimulants, but not nonstimulant ADHD medications, reduced suicide attempts across ADHD patients of all ages, including children, adolescents, and young adults, and in patients with co-occurring depression and substance use disorders. A retrospective analysis [210] found that methylphenidate reduced suicide attempts in under-18 youth with ADHD, with greater reductions in risk being associated with longer duration of treatment. To determine whether reduction in impulsivity mediates these reductions in suicide risk, and whether stimulant medications can target impulse control deficits occurring in negative emotional states, will require further research and prospective clinical trials.

### Identity/self-processing interventions

Systematic psychotherapeutic interventions and somatic treatments focused on adolescents’ identity (e.g., promoting a more flexible and positive self-view) are lacking. This highlights an area of critical need for teens whose STBs are associated with negative self-evaluation or diffuse identity development. Engaging brain targets implicated in self-processes, such as cingulate, medial prefrontal, and ventromedial cortices and the DMN, may yield treatments especially applicable to behaviors that intersect with adolescent identity development. However, additional research is needed both to examine modulation of self-processes in reducing STBs and to understand neural systems engaged in such therapies.

### Social development interventions

Interpersonal psychotherapy is another well-established treatment for depression in adolescents which directly focuses on improving social relationships [211]. Interpersonal psychotherapy has been shown to reduce suicidal ideation in adolescents [212] and has been adapted specifically for NSSI [213]. In a pilot study of neural predictors of interpersonal psychotherapy response in adolescents, Klimes-Dougan and colleagues [214] found that greater ACC activation during an emotion-matching task and greater amygdala–ACC resting-state functional connectivity were related to greater improvement in depressive symptoms. Although STBs were not the focus of this study, nor was randomization across other treatments examined, this provides preliminary evidence that imaging markers of neural systems involved in threat-assessment and emotion regulation processes may help identify adolescents likely to benefit from interpersonal psychotherapy.

### Synergistic interventions and novel approaches

The structure of neuromodulatory intervention delivery provides opportunities to integrate psychotherapy and behavioral interventions. Practically, there are often lost opportunities in the delivery of neuromodulation treatments, as psychotherapeutic approaches could be delivered concurrently or in close succession. This integration also provides opportunities to understand brain mechanisms, enhance therapeutic effectiveness via priming or consolidation effects of stimulation, and improve the rigor of clinical trials with normalized brain states across research participants. Furthermore, prospective studies targeting brain systems involved in psychotherapies focused on developmental tasks (e.g., distress tolerance skills, cognitive control training, reducing self-referential rumination, and interpersonal coaching) are needed to establish whether TMS can reduce STBs via enhancing plasticity for these processes.

## Considerations for future research directions

### Neural mechanisms for the range of and transitions between STBs

The broad range of emotions, thoughts, and behaviors related to suicide that occur in adolescents do not represent a single, unidimensional spectrum. Accordingly, while some commonalities in their neurodevelopmental underpinnings are apparent, the literature also suggests that different types and severities of STBs that evolve over adolescent development may have distinct neural signatures. The transition between suicidal thoughts and actions may involve yet other neural mechanisms, which remain poorly understood [215]. Although trait impulsivity has been posited as a factor in this pivot from ideation to attempt, this is not broadly supported in the literature [215–217]. Diminished impulse control during negative mood states has been linked to the progression of suicidal ideation to behavior in adults [217], but further research across the adolescent age span will yield insights into developmental neural mechanisms of negative urgency in relation to risk of suicide attempts. Contemporary theoretical models for the ideation-to-attempt transition include the Interpersonal-Psychological Theory [140], Three-Step Model [218, 219], Integrated Motivational-Volitional Model [220, 221], and Fluid Vulnerability Theory [222]. These theories have significant overlap in emphasizing the roles of pain, defeat, and hopelessness in creating suicidal desire; lack of interpersonal belonging to deter suicidal action; acquiring the capability to carry out suicidal actions in spite of inhibitions; and they acknowledge that the confluence of these factors is dynamic over time [223]. Yet, as Kirshenbaum et al. [224] recently acknowledged in their review, these theories have not been tested extensively in adolescent populations; unique biological and social factors of the teenage years, as well as particular risks in certain populations, may necessitate developmental and cultural refinement of these theories for adolescent applications. Future research must include youth with a broad, diverse range of STBs and have sufficient timescales to assess ideation-to-attempt transitions in order to enhance our understanding of developmental mechanisms of risk escalation. Studying adolescents with higher clinical acuity, such as those with recent attempts, is challenging from standpoints of recruitment, ethics, and safety in typical prospective study designs; yet, it is critical to include teens at high risk to understand the neurobiology of acute suicidal states.

### Emerging methods for studying youth STBs

Although our review focuses on neuroimaging, primarily fMRI, other methodologies and study designs offer unique opportunities to study suicidal teens. A limited number of postmortem studies have been conducted in adolescents who died by suicide. Prior work examining adolescent suicide decedents’ brain tissue found brain-derived neurotrophic factor (BDNF) transcription and binding alterations, as well as elevated inflammatory cytokines, in the PFC [225–228]. Electroencephalography research has pointed to other processes that may be involved in suicide risk in youth, such as attentional bias to self-relevant stimuli [229, 230], attentional control [231, 232], and emotional response inhibition [233]. Building on recent approaches using multiple units of analysis (across experiential, physiological, and neural levels) that suggest a unique signature for discordant stress responses in youth with NSSI [234, 235], multimodal methods may have promise for identifying similar or distinct patterns in adolescents with STBs.

Longitudinal study designs also are critical for observing developmental brain changes and potential shifts in neural functions that occur with the onset of suicidal ideation and the progression from ideation to action. Although the ABCD Study [49] is currently in progress, analyses from early timepoints (e.g., [236]) are emerging. The utility of these data in elucidating developmental neural trajectories of STBs is only partially realized due to low base rates of attempts and suicide deaths in community samples, particularly during early adolescence. However, future analyses using the ABCD Study’s dataset will benefit not only from its large sample but also its long observational period from emerging adolescence to emerging adulthood, a critical age range for ideation-to-attempt transitions [28].

Identification of reliable biomarkers for suicide risk has proven elusive, despite decades of research [237], particularly for biological factors that predict future behavior [238]. Even prior NSSI and STBs show reasonable specificity but poor sensitivity in prediction [15]. Precision medicine approaches that individualize risk assessment and treatment have been explored in suicidology. Prior work has identified genetic, gene expression, and epigenetic markers of stress response and neuroinflammation implicated in suicide risk, which could be assessed quantitatively to devise treatment patient-specific treatment based on an individual’s unique biological profile [239]. One promising approach has been the use of machine learning models that incorporate large numbers of variables to predict risk of future suicidal events at an individual level (for reviews, see [240, 241]). However, most machine learning approaches for prediction of STBs, including those in adolescent studies [242, 243], have relied on demographic and clinical characteristics and survey-based information, not imaging or other biological data [241]. The addition of neuroimaging and other neurophysiologic data could offer powerful input into machine learning methods for risk prediction [239], yet a more feasible approach may involve multiple tools that together serve as proxies for neurobiological systems. Wearable technologies and smartphone-based measures for ecological momentary assessment (EMA) of emotional and physiologic factors that fluctuate on brief timescales, coupled with computational approaches for such temporally-dense data, are practical for use in teens, and are increasingly available [244]. The feasibility of tracking behavioral and physiological changes, as well as suicidal ideation, longitudinally via smartphone-based applications has been demonstrated in high-risk adolescent samples [245]. Both active (brief surveys) and passive (actigraphy, location data, and engagement with social media, calls, and text messages) data collection have been examined for suicide risk assessment and monitoring [246]. Furthermore, a combination of traditional survey and EMA data may offer complimentary but distinct measures that correspond to neuroimaging and other biological metrics, providing both static (trait) and dynamic (state) views of constructs relevant to STBs [129]. Inclusion of measures that assess multiple facets of a particular characteristic, symptom, or behavior pertaining to suicide risk may further improve the ability of computational approaches to yield clinically-significant, individual-level prediction.

### Need for global and representative research

A notable limitation of the adolescent brain development literature, particularly research using neuroimaging methods, is that most evidence has been collected in high-income countries. Although some elements of neural development related to emotion regulation, impulse control, self-conceptualization, and social interactions are likely universal, there may be different developmental trajectories related to differences in environmental stressors experienced by adolescents in low-, middle-, and high-income nations [247]. Psychosocial stressors are overrepresented in youth who drop out of longitudinal studies [248], yet retention is critical for generalizable findings. Recent endeavors have focused on using multimodal methods, including neuroimaging, for early detection of youth at elevated risk for depression in diverse socioeconomic conditions across the world [249, 250]. Similar efforts are needed to gather data about risk factors for adolescent STBs. Increased research in less affluent countries, where youth suicide attempt rates are even higher than in high-income nations [27], will improve the generalizability of findings on neural processes involved in suicide risk in youth.

## Conclusions

We posit that the neurobiological underpinnings of the emergence of suicidal thoughts and behaviors in adolescence can be understood in the context of the critical yet complex developmental tasks of the adolescent years. The processes through which adolescents experience and regulate new and often intense emotions, learn to inhibit impulses and regulate behavior, refine their identities, and forge new social relationships permit responsiveness to teens’ environments, and are key to achieving independence and the skills necessary for the transition to adulthood. Brain systems underlying these critical developmental tasks and the dramatic changes observed in affect, behavior, and relationships during adolescence have been studied in typically-developing youth and in those with various forms of psychopathology. Although neural plasticity creates opportunities for tremendous growth and adaptability to changing environments, it can create vulnerabilities. Emerging evidence suggests that youth with STBs show important disruptions in specific affective, impulse-control, self, and social processes that are conceptually linked to suicide risk. Adolescents who experience STBs have changes in brain structure and functioning that undergird these key developmental processes, particularly in medial prefrontal and other midline cortical structures as well as the limbic system. Precision medicine and machine learning approaches, large-sample multimodal datasets with longitudinal observation, and dense ecological experience sampling will further elucidate neural mechanisms of these processes, and these emerging insights must be leveraged in conceptualizing novel interventions designed to reduce suicide risk. Developmentally-targeted treatments that engage the neural infrastructure of the emotional–cognitive–social tasks of adolescence hold not merely the promise of enhancing teens’ survival; such interventions will restore developmental trajectories and help young people thrive throughout adulthood. Further research into neurobiological mechanisms of STBs will yield more effective, developmentally-specific tools for predicting suicidal behavior and for deploying technological advances to reduce the risk for suicide.
